# Development of a well-defined tool to predict the overall survival in lung cancer patients: an African based cohort

**DOI:** 10.1186/s12885-023-11355-7

**Published:** 2023-10-20

**Authors:** Hassan Abdelilah Tafenzi, Farah Choulli, Ganiou Adjade, Anas Baladi, Leila Afani, Mohammed El Fadli, Ismail Essaadi, Rhizlane Belbaraka

**Affiliations:** 1 Medical Oncology Department, Mohammed VI University Hospital of Marrakech, Marrakech, Morocco; 2 Faculty of Medicine and Pharmacy, Biosciences and Health Laboratory, Cadi Ayyad University, Marrakech, Morocco; 3 Medical Oncology Department, Avicenna Military Hospital of Marrakech, Marrakech, Morocco

**Keywords:** Lung cancer, Overall survival, Nomogram

## Abstract

**Background:**

Nomogram is a graphic representation containing the expressed factor of the mathematical formula used to define a particular phenomenon. We aim to build and internally validate a nomogram to predict overall survival (OS) in patients diagnosed with lung cancer (LC).

**Methods:**

We included 1200 LC patients from a single institution registry diagnosed from 2013 to 2021. The independent prognostic factors of LC patients were identified via cox proportional hazard regression analysis. Based on the results of multivariate cox analysis, we constructed the nomogram to predict the OS of LC patients.

**Results:**

We finally included a total of 1104 LC patients. Age, medical urgency at diagnosis, performance status, radiotherapy, and surgery were identified as prognostic factors, and integrated to build the nomogram. The model performance in predicting prognosis was measured by receiver operating characteristic curve. Calibration plots of 6-, 12-, and 24- months OS showed optimal agreement between observations and model predictions.

**Conclusion:**

We have developed and validated a unique predictive tool that can offer patients with LC an individual OS prognosis. This useful prognostic model could aid doctors in making decisions and planning therapeutic trials.

## Background

Lung cancer (LC) remains the most lethal type of cancer worldwide and in Morocco as well, accounting for 85% of all diagnosed Non-Small Cell Lung Cancer (NSCLC) and 13%-14% of Small Cell Lung Cancer (SCLC), with 1% of other histology types [1]. Growing evidence suggests that smoking is the major risk factor related to lung cancer, causing deregulated molecular pathways and or a specific type of mutations in a specific genome.

For early LC stages, including stage I, II, and III, the standard curative treatment is chemotherapy in association with radiotherapy, and if indicated, the patient may undergo local or radical resection. Patients with non-metastatic LC are categorized on the basis of tumor size, and invasion as well as the level of lymph node involvement, according to the eighth edition of the American Joint Committee on Cancer TNM classification [2]. Patients with the same stage of cancer have a wide range of survival rates. It is thought that various stages of LC are influenced by different prognostic factors such as age, smoking status, gender, histological type, invasion of tumor size, nodal status, and treatment-related factors, all of which could significantly play a role in individualized prediction survival [3, 4]. Indeed, because evidence suggests that tumor size and N stage are strongly related to the biological characteristic of the tumor, and because they are based on tumor depth invasion, they remain the most important tumor characteristics, and are therefore considered a robust risk factor for LC survival [5–8].

Various models have been developed and widely accepted as reliable tools to quantify risks, and predict survival by integrating and using key elements for oncological prognosis [9–11]. However, dating back to the work of Thomlinson & Gray (1955), the first mathematical model in oncology fields was proposed for the avascular tumor growth of LC by demonstrating that the size of the observed histological pattern is consistent with what would be predicted if oxygen supply were the limiting factor determining the onset of necrosis [12]. Based on multifactorial regression analysis, the prognosis of outcome differs from the used approach, and tool as well. In fact, the combination of multiple predictive factors to build and validate an individual prognostic tool such as nomogram, makes the results more reliable [13, 14], and accessible in terms of patient prognosis [15].

In this study, we aim to build and validate a comprehensive prognostic evaluation system for LC patients based on multiple clinical and pathological prognostic inputs hoping to provide more reliable predictions.

## Methods

### Patients’ selection and data elements

A single-institution registry consisting of 1200 patients has been diagnosed with Lung Cancer between January 2013 and December 2021 from the Medical Oncology Department of the Mohammed VI University Hospital of Marrakesh, Morocco was established. To retrieve all essential data, standardized LC patients’ confirmed pathological characteristics including Age at diagnosis, Gender, Tabaco status, Cannabis status, Alcohol, Body Weight, Performance Status; Presence or absence of urgency at diagnosis including Superior Vena Cava Syndrome (SVCS) or Pleurisy syndrome; Comorbidities; Clinic-pathologic data including Pathological T, N, M categories, presence or absence of Liver Metastasis, Adrenal Metastasis, Bone Metastasis, and Brain Metastasis, Stage at diagnosis; EGFR, ALK, PDL-1; treatment-related data including Surgery, Chemotherapy, Radiotherapy; hematological toxicities reported during treatment including Anemia, Neutropenia, Thrombocytopenia; form was established. All patients' follow-up information was extracted from their most recent medical review, which included a clinical examination and/or a review of computed tomography images. The eighth edition of the American Joint Committee on Cancer TNM classification system was used to determine pathological staging. Age and weight, as continuous variables, were transformed into a categorical variables based on quartiles. Weight is defined as body mass at diagnosis and is reflected by the unit of kilograms (Kg).

We define tobacco consumption as smoking cigarettes, whereas smoking marijuana is the definition of cannabis consumption. Due to the retrospective nature of the study, the exact quantity is not mentioned in all patient medical records, thus we could not define either patient is a heavy or light smoker.

Variables with more than 20% of missing values were excluded. In addition, patients were also excluded from the subsequent analysis if they missing important detail information such date of biopsy or survival date, information on treatments such as radiotherapy, chemotherapy, or surgery. Finally, 1104 eligible identified LC cases were selected for the study.

The main objective element of this paper was OS, which was defined as the interval time between the biopsy day to death without specific cause.

### Statistical analysis

All LC patients were randomly assigned (*n=*730) for training and (*n=*374) for validation cohorts with a 2:1 ratio. Categorical variables were expressed as percentages. In the training cohort, a univariate cox analysis was performed to determine the variables related to prognosis. Then, the independent prognostic variables related to the OS of LC patients were determined using multivariate cox analysis, where only factors with a *p*-value less than 0.05 are considered statistically significant and were therefore incorporated to develop the nomogram.

Due to the necessity to test the reliability of the model, four key elements were established to assess the results performance of prediction probabilities for 6, 12, and 24 months. First, a 300 bootstrap resampling method was adopted to internally validate the nomogram. Second, the calibration curve was plotted to compare the consistency of projected clinical responses probability versus actual response proportion, which should be close to 45 degrees. Third, the area under the time-dependent receiver operating characteristic (ROC) was adopted to assess the discrimination. Fourth, the C-Index was used to judge the model’s prediction accuracy, given the closer C-index to value 1, the greater precision is [16].

Survival curves for sex, age at diagnosis, medical urgency at diagnosis, PS, radiotherapy, and surgery values were generated using the Kaplan-Meier estimates. The log-rank test was adopted to compare the subgroups of these variables, as reflected by the *p*-value; the smaller the *p*-value, the greater the difference.

All statistical analyses to identify the independent prognostic factors and to build the model were performed using R-software version 4.1.3. Available from: http://www.r-project.org) with “survival”, survminer”, and “rms” [17] packages.

## Results

### Patients’ characteristics

Based on selected criteria, the 1104 enrolled LC patients’ characteristics cases, divided into training (*n=*730) and validation (*n=*374) cohorts, are summarized in Table 1. We should note the significant absence of differences among these cohorts. In the training set, the vast majority of patients were male (*n=*654), diagnosed above 66 years old, and most of them died during treatment. Meanwhile, in terms of tumor characteristics, LC patients were often diagnosed at advanced T4, and N2 stages, M1b and (27.3%) with bone metastasis, followed by brain, adrenal, and liver metastasis at diagnosis. Most of the patients were diagnosed at late stage IVA ( *n =* 448, 61,4%) and IVB (*n =* 212, 29%). Moreover, adenocarcinoma was the most appearing histological type (49.1%), and SVCS (5.3%) was the most present urgency at diagnosis. As for treatment, most of patients had not received radiation therapy (86.6%), and surgery (95.2%), but most of them received chemotherapy (57.1%). Regarding hematological toxicities reported during treatment, most patients did not report anemia, neutropenia, or thrombocytopenia with (21.3%), (35.5%), and (39.9%), respectively.

**Table 1:** Demographic, clinic, pathologic characteristics for LC patients in training and validation cohorts

	***Training cohort***		***Validation cohort***	
	***N=730***		***N = 374***	
***Characteristics***	***n***	***%***	***n***	***%***
Sex				
Female	*76*	*10.40%*	*47*	*12.60%*
Male	*654*	*89.60%*	*327*	*87.40%*
Age at diagnosis				
20-54	*174*	*23.90%*	*94*	*25.10%*
55-60	*185*	*25.30%*	*92*	*24.60%*
61-66	*158*	*21.60%*	*69*	*18.50%*
> 66	*212*	*29.10%*	*119*	*31.80%*
Missing Values	*1*	*0.10%*	*0*	*0%*
Tabaco				
No	*123*	*16.90%*	*71*	*19%*
Yes	*601*	*82.30%*	*295*	*78.90%*
Missing Values	*6*	*0.80%*	*8*	*2.10%*
Cannabis				
No	*612*	*83.90%*	*313*	*83.70%*
Yes	*112*	*15.30%*	*54*	*14.40%*
Missing Values	*6*	*0.80%*	*7*	*1.90%*
Alcohol				
No	*602*	*82.50%*	*313*	*83.70%*
Yes	*122*	*16.70%*	*54*	*14.40%*
Missing Values	*6*	*0.80%*	*7*	*1.90%*
Comorbidities				
NO	*557*	*76.30%*	*302*	*80.80%*
Cancer	*8*	*1.10%*	*4*	*1.10%*
Cardiac	*33*	*4.50%*	*13*	*3.50%*
Endocrine	*42*	*5.70%*	*12*	*3.20%*
Pulmonary	*46*	*6.30%*	*20*	*5.30%*
Family	*13*	*1.80%*	*11*	*2.90%*
Surgical	*29*	*4%*	*11*	*2.90%*
Missing Values	*2*	*0.30%*	*1*	*0.30%*
Histology				
ADK	*358*	*49.10%*	*199*	*53.20%*
ASCC	*5*	*0.70%*	*4*	*1.10%*
EC	*168*	*23%*	*75*	*20%*
NEC	*43*	*5.90%*	*22*	*5.90%*
SCC	*33*	*4.50%*	*18*	*4.80%*
Missing Values	*123*	*16.80%*	*56*	*15%*
EGFR				
Wild type	*46*	*6.30%*	*11*	*2.90%*
Mutant	*10*	*1.40%*	*7*	*1.80%*
Missing Values	*674*	*92.30%*	*356*	*95.30%*
ALK				
Expressed	*2*	*0.20%*	*1*	*0.20%*
Not Expressed	*25*	*3.40%*	*18*	*4.80%*
Missing Values	*703*	*96.40%*	*355*	*95%*
PDL-1				
< 1%	*25*	*3.40%*	*7*	*1.90%*
1% - 49%	*7*	*0.90%*	*5*	*1.40%*
≥ 50%	*5*	*0.70%*	*2*	*0.60%*
Missing Values	*693*	*95%*	*360*	*96.10%*
T Clinical category				
I	*35*	*4.80%*	*20*	*5.30%*
II	*104*	*14.20%*	*50*	*13.40%*
III	*135*	*18.50%*	*86*	*23%*
IV	*397*	*54.40%*	*183*	*48.90%*
Missing Values	*59*	*8.10%*	*35*	*9.40%*
N Clinical category				
N_0_	*97*	*13.30%*	*38*	*10.10%*
N_1_	*151*	*20.70%*	*84*	*22.50%*
N_2_	*328*	*44.90%*	*179*	*47.90%*
N_3_	*106*	*14.50%*	*44*	*11.80%*
Missing Values	*48*	*6.60%*	*29*	*7.70%*
M Clinical category				
0	*46*	*6.30%*	*17*	*4.60%*
1a	*221*	*30.30%*	*129*	*34.50%*
1b	*227*	*31.10%*	*122*	*32.60%*
1c	*212*	*29%*	*88*	*23.50%*
Missing Values	*24*	*3.30%*	*18*	*4.80%*
Liver Metastasis				
No	*620*	*84.90%*	*314*	*84%*
Yes	*96*	*13.20%*	*46*	*12.30%*
Missing Values	*14*	*1.90%*	*14*	*3.70%*
Adrenal Metastasis				
No	*573*	*78.50%*	*302*	*80.80%*
Yes	*137*	*18.80%*	*54*	*14.40%*
Missing Values	*20*	*2.70%*	*18*	*4.80%*
Bone Metastasis				
No	*511*	*70%*	*256*	*68.50%*
Yes	*199*	*27.30%*	*100*	*26.70%*
Missing Values	*20*	*2.70%*	*18*	*4.80%*
Brain Metastasis				
No	*556*	*76.20%*	*285*	*76.20%*
Yes	*160*	*21.90%*	*83*	*22.20%*
Missing Values	*14*	*1.90%*	*6*	*1.60%*
Stage at diagnosis				
IA	*4*	*0.50%*	*0*	*0%*
IIA	*1*	*0.10%*	*5*	*1.30%*
IIB	*9*	*1.20%*	*2*	*0.60%*
IIIA	*29*	*4.10%*	*10*	*2.70%*
IIIB	*1*	*0.10%*	*0*	*0%*
IVA	*448*	*61.40%*	*251*	*67.10%*
IVB	*212*	*29%*	*88*	*23.50%*
Missing Values	*26*	*3.60%*	*18*	*4.80%*
Urgencies				
No	*599*	*82.10%*	*310*	*82.90%*
SVCS	*39*	*5.30%*	*23*	*6.10%*
Pleurisy Syndrome	*83*	*11.40%*	*37*	*9.90%*
Missing Values	*9*	*1.20%*	*4*	*1.10%*
PS(OMS)				
1	*346*	*47.40%*	*187*	*50%*
2	*196*	*26.80%*	*89*	*23.80%*
3	*95*	*13%*	*47*	*12.60%*
4	*32*	*4.40%*	*14*	*3.70%*
Missing Values	*61*	*8.40%*	*37*	*9.90%*
Radiotherapy				
No	*632*	*86.60%*	*321*	*85.80%*
Yes	*98*	*13.40%*	*53*	*14.20%*
Chemotherapy				
No	*313*	*42.90%*	*161*	*43%*
Yes	*417*	*57.10%*	*213*	*57%*
Surgery				
No	*695*	*95.20%*	*358*	*95.70%*
Yes	*35*	*4.80%*	*15*	*4%*
Missing Values	*0*	*0%*	*1*	*0.30%*
Anemia				
G0	*155*	*21.30%*	*65*	*17.30%*
G1	*79*	*10.90%*	*37*	*10%*
G2	*73*	*10%*	*44*	*11.80%*
G3	*39*	*5.40%*	*26*	*6.90%*
G4	*4*	*0.60%*	*5*	*1.30%*
Missing Values	*378*	*51.80%*	*197*	*52.70%*
Neutropenia				
G0	*259*	*35.50%*	*130*	*34.80%*
G1	*45*	*6.20%*	*16*	*4.30%*
G2	*13*	*1.80%*	*9*	*2.40%*
G3	*20*	*2.70%*	*11*	*2.90%*
G4	*14*	*1.80%*	*13*	*3.50%*
Missing Values	*379*	*52%*	*195*	*52.10%*
Thrombocytopenia				
G0	*291*	*39.90%*	*146*	*39%*
G1	*22*	*3%*	*11*	*2.90%*
G2	*12*	*1.60%*	*7*	*1.90%*
G3	*10*	*1.40%*	*6*	*1.60%*
G4	*15*	*2%*	*7*	*1.90%*
Missing Values	*380*	*52.10%*	*197*	*52.70%*
Weight				
<55	*110*	*15.10%*	*72*	*19.30%*
55-61	*70*	*9.60%*	*35*	*9.40%*
>61	*52*	*7.10%*	*20*	*5.30%*
Missing Values	*498*	*68.20%*	*247*	*66%*

### Survival analysis

**Fig. 1:**
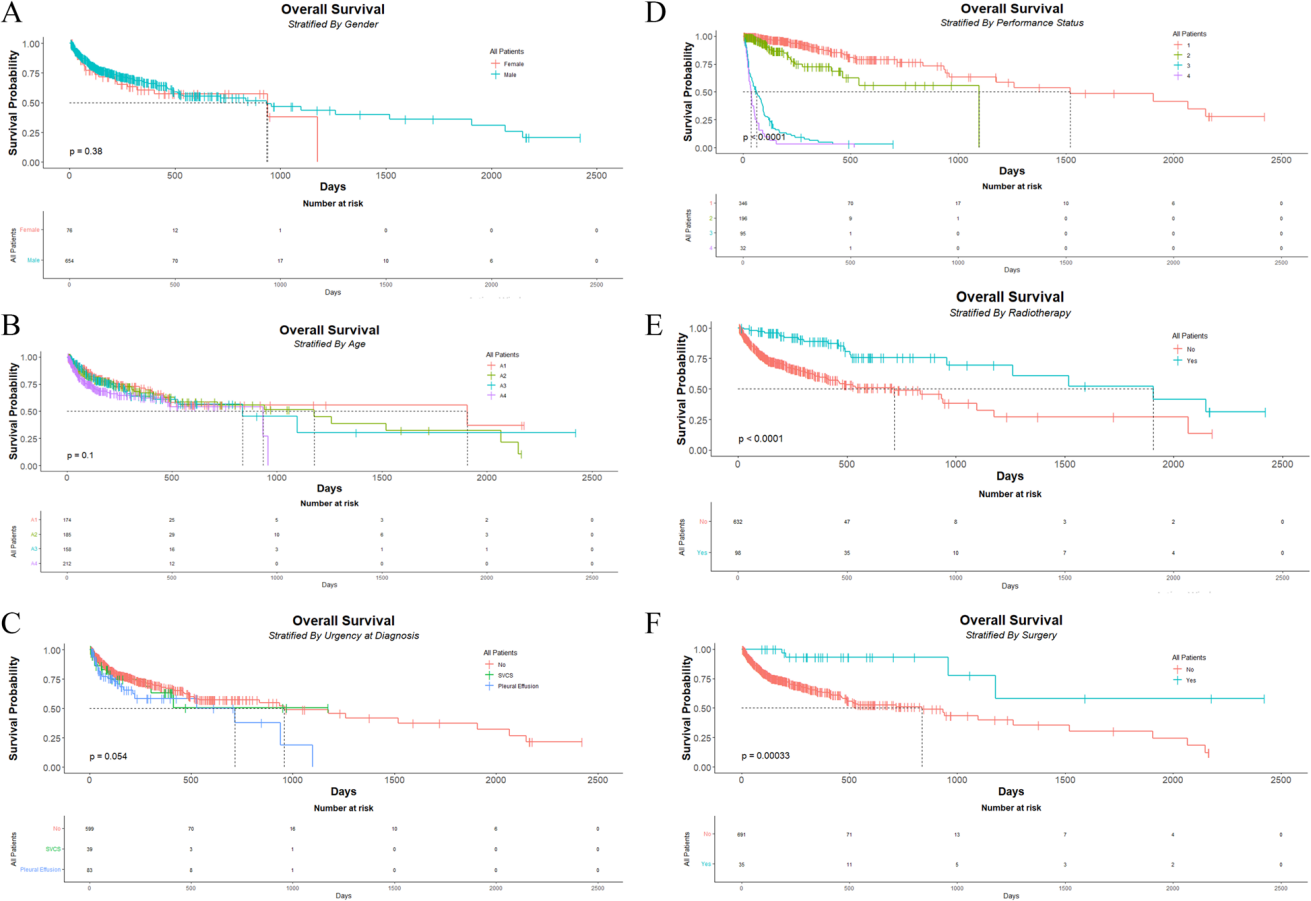
Kaplan-Meir curves stratified by : **A**- Gender, **B**- Age, **C**-Medical Urgency at Diagnosis, **D**- Performance Status, **E**- Radiotherapy, **F**- Surgery

Figure 1 presents the differences in survival between the subgroups, involving radiotherapy, age at diagnosis, urgency at diagnosis, and surgery. The median OS for the entire cohort was 934 (95% CI: 634, 1176) days. In total, 291 deaths were registered.

### Independent prognostic factors

The following variables have been subjected to univariate Cox analysis (UNCA): sex, age, Tabaco smoking, cannabis smoking, alcohol, comorbidities, histology type, T stage, N stage, M stage, liver metastasis, adrenal metastasis, bone metastasis, brain metastasis, medical urgency at diagnosis, PS, weight, chemotherapy, radiotherapy, surgery, anemia, neutropenia, and thrombocytopenia. The results of UVCA showed that age, comorbidities, M stage, brain metastasis, medical urgency at diagnosis, PS, weight, radiotherapy, chemotherapy, surgery, and anemia were prognostic factors for LC patients (Table 2). These UVCA results were subsequently interred in a multivariate Cox analysis (MVCA). Finally, 5 factors were identified as independent prognostic ones including: age, medical urgency at diagnosis, PS, radiotherapy, and surgery.

**Table 2:** Univariate and multivariate cox regression analysis of prognosis for LC patients

	***Univariate Cox Analysis***		***Multivariate Cox Analysis***	
***Characteristics***	***HR (95% CI)***	***p-value***	***HR(95% CI)***	***p-value***
Sex				
Female	*Reference*			
Male	*0.83(0.55, 1.26)*	*0.4*		
Age at diagnosis				
20-54	*Reference*			
55-60	*1.13(0.76, 1.69)*	*0.6*	*3.61(1.09, 12.0)*	*0.03*
61-66	*1.14(0.74, 1.74)*	*0.6*	*3.64(0.95, 13.9)*	*0.06*
> 66	*1.59(1.07, 2.36)*	*0.02*	*0.20(0.03, 1.53)*	*0.12*
Tabaco				
No	*Reference*			
Yes	*1.09(0.75, 1.57)*	*0.6*		
Cannabis				
No	*Reference*			
Yes	*1.04(0.71, 1.53)*	*0.8*		
Alcohol				
No	*Reference*			
Yes	*0.99(0.69, 1.42)*	*0.9*		
Comorbidities				
NO	*Reference*			
Cancer	*0.98(0.31, 3.11)*	*0.9*		
Cardiac	*1.8(1.04, 3.12)*	*0.036*		
Endocrine	*0.77(0.39, 1.52)*	*0.5*		
Pulmonary	*0.95 (0.52, 1.76)*	*0.9*		
Family	*2.47(1.09, 5.60)*	*0.031*		
Surgical	*1.07(0.53, 2.19)*	*0.8*		
Histology				
ADK	*Reference*			
ASCC	*0.80 (0.11, 5.74)*	*0.8*		
EC	*0.9(0.62, 1.29)*	*0.6*		
NEC	*1.00(0.55, 1.82)*	*0.9*		
SCC	*1.69(0.88, 3.24)*	*0.12*		
T Clinical category				
I	*Reference*			
II	*2.55 (0.90, 7.23)*	*0.078*		
III	*1.41(0.49, 4.05)*	*0.5*		
IV	*2.53(0.93, 6.88)*	*0.068*		
N Clinical category				
N_0_	*Reference*			
N_1_	*0.71(0.42, 1.22)*	*0.2*		
N_2_	*1.08(0.68, 1.69)*	*0.8*		
N_3_	*1.57(0.94, 2.63)*	*0.087*		
**M Clinical category**				
**0**	***Reference***			
**1a**	***1.59(0.76, 3.32)***	***0.2***		
**1b**	***1.65(0.78, 3.47)***	***0.2***		
**1c**	***2.14(1.02, 4.48)***	***0.04***		
Liver Metastasis				
No	*Reference*			
Yes	*1.42(0.95, 2.11)*	*0.085*		
Adrenal Metastasis				
No	*Reference*			
Yes	*1.37(0.96, 1.95)*	*0.086*		
Bone Metastasis				
No	*Reference*			
Yes	*1.03(0.75, 1.43)*	*0.8*		
Brain Metastasis				
No	*Reference*		*Reference*	
Yes	*1.54(1.11, 2.12)*	*0.009*	*0.34(0.08, 1.44)*	*0.14*
Urgencies				
No	*Reference*		*Reference*	
SVCS	*1.22 (0.66, 2.25)*	*0.5*	*13.3(2.65, 66.7)*	*0.002*
Pleurisy Syndrome	*1.64 (1.09, 2.47)*	*0.018*	*3.81(0.94, 15.5)*	*0.06*
PS(OMS)				
1	*Reference*		*Reference*	
2	*2.43(1.47, 4.01)*	*<0.001*	*2.96 (0.87, 10.1 )*	*0.08*
3	*26.7 (17.7, 40.3)*	*<0.001*	*52.7 (8.28, 336)*	*<0.001*
4	*45.3(27.4, 74.9)*	*<0.001*	*NA*	*<0.001*
Radiotherapy				
No	*Reference*			
Yes	*0.32(0.20, 0.52)*	*<0.001*	*0.1(0.02, 0.47)*	*0.003*
Chemotherapy				
No	*Reference*			
Yes	*0.11(0.08, 0.16)*	*<0.001*	*0.52(0.04, 7.01)*	*0.6*
Surgery				
No	*Reference*			
Yes	*0.19(0.07, 0.53)*	*0.001*	*0.08(0.01, 0.61)*	*0.01*
Anemia				
G0	*Reference*			
G1	*1.60(0.84, 3.03)*	*0.2*	*0.53(0.11, 2.45)*	*0.4*
G2	*1.41(0.68, 2.91)*	*0.4*	*0.59(0.18, 1.99)*	*0.4*
G3	*1.41(0.64, 3.10)*	*0.4*	*1.13(0.24, 5.39)*	*0.9*
G4	*5.29(1.20, 23.2 )*	*0.027*	*2.48(0.22, 27.5)*	*0.5*
Neutropenia				
G0	*Reference*			
G1	*0.70 (0.28, 1.78)*	*0.5*		
G2	*0.73(0.23, 2.36)*	*0.6*		
G3	*0.65(0.20, 2.11)*	*0.5*		
G4	*0.84(0.20, 3.48)*	*0.8*		
Thrombocytopenia				
G0	*Reference*			
G1	*0.68(0.16, 2.81)*	*0.6*		
G2	*0.0(0.0, 0.0)*	*>0.9*		
G3	*1.17(0.36, 3.78)*	*0.8*		
G4	*2.7 (0.96, 7.61)*	*0.06*		
Weight				
<55	*Reference*			
55-61	*0.57(0.29, 1.12)*	*0.1*	*1.30(0.39, 4.28)*	*0.7*
>61	*0.4(0.17, 0.93)*	*0.034*	*0.88(0.25, 3.11)*	*0.8*

### Prognostic nomogram for OS

**Fig. 2:**
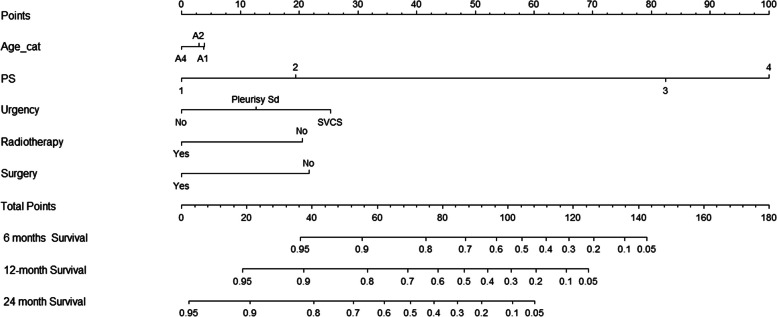
Nomogram predicting 6-, 12-, and 24- months OS. The total points were calculated by adding the points of each prognostic factor, and correspond to the possibilities of 1-year, 2-year, and 3-year OS of LC patients. Sd = Syndrome, SVCS = Superior Vena Cava Syndrome, OS = overall survival

The independent prognostic factors derived from the MVCA were used to build the nomogram to predict the OS for LC patients (Fig. 2). As shown in Fig. 2, performance status and medical urgency at diagnosis have the greatest contribution to prognosis, followed by radiotherapy, and surgery with the same moderate impact on prognosis, while age at diagnosis has the minimal effect on prognosis. Each variable subtype assigned a score on the point scale. We were easily able to draw a straight line down to determine the expected likelihood of survival at each time point by adding up the total score and projecting it onto the total point scale.

### Evaluation of nomogram

**Fig. 3:**
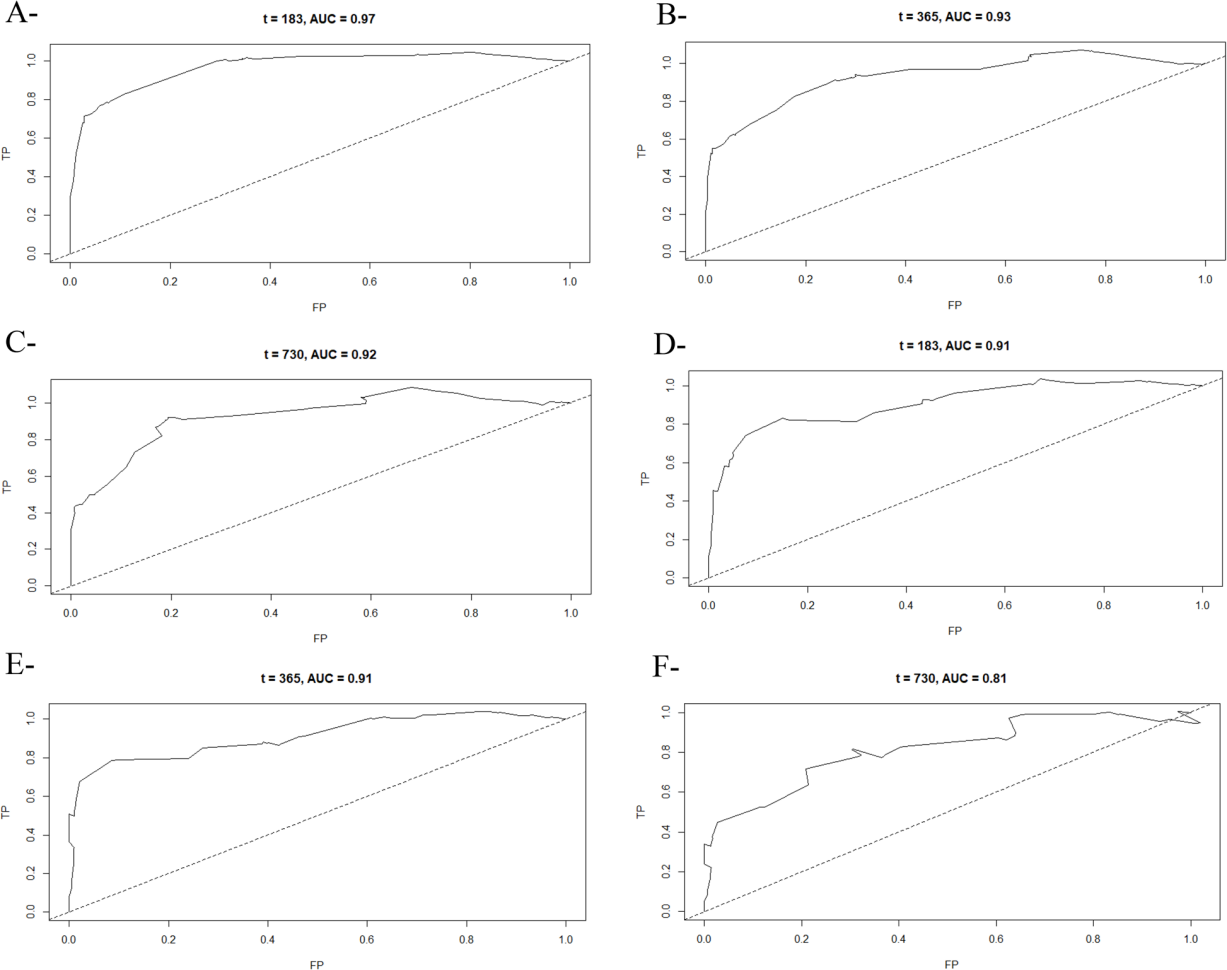
AUROC Curves of training (**A**-**B**-**C**) and Validation (**D**-**E**-**F**) set of the Nomogram for predicting 6-months, 12-months, and 24-months OS

**Fig. 4:**
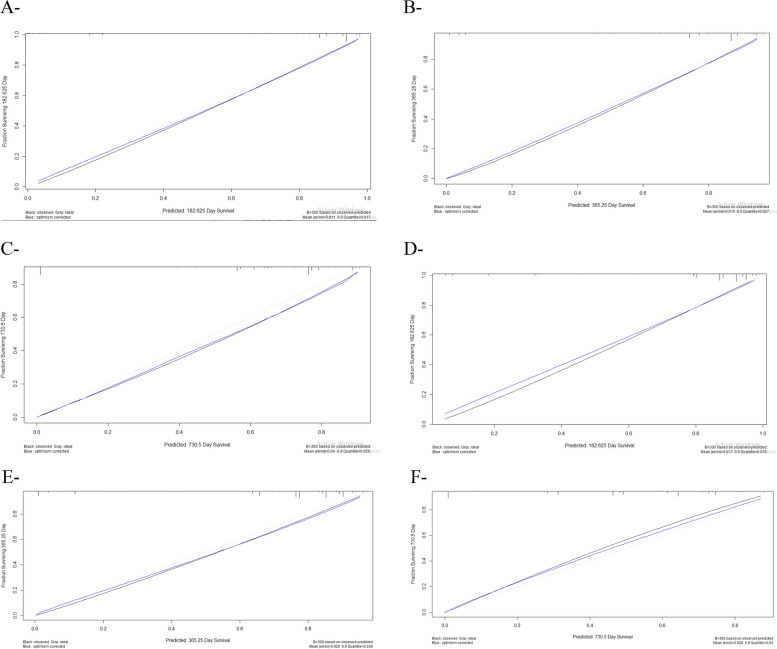
Calibration plot for training (**A**-**B**-**C**) and validation data (**D**-**E**-**F**)

The ROC plots showed that the AUC of the clinical predictive model for 6-, 12-, and 24- months OS scored 0.97, 0.93, 0.92 in the training set, and 0.91, 0.91, 0.81, in the validation set respectively, demonstrating a better discriminative ability (Fig. 3). Furthermore, the calibration plots for 6-, 12-, and 24- months OS showed an excellent agreement in both, the primary and validation cohorts between observed probabilities and nomogram predicted probabilities (Fig. 4). Stratification into different subgroups demonstrates a distinction between Kaplan-Meier curves for LC patients’ prognosis.

## Discussion

Due to the heterogeneity related to individual LC patients [18], predicting survival using demographic, clinic, biologic, and pathologic characteristics is imprecise. Several prognostic models have been developed and discussed based on a specific cohort and outcome, but no nomogram has been constructed based on a purely well-defined African cohort. Thus, we sought to establish a convenient predictive model based on 1104 enrolled cases with 5 independent prognostic factors identified by Cox regression analysis to predict 6-, 12-, and 24- months OS of LC patients.

The data were extracted and collected manually from the registry of a single public institution. This institution is the only leading public medical center representing central and southern Morocco, and contains all the standard technical care accepted in the kingdom.

We found, in this research, through a subsequent multivariate Cox analysis that age, medical urgency at diagnosis, performance status, surgery, and radiotherapy were the prognostic factors related to progression, which were consistence with previously reported results [19–27]. The integration of various clinical, pathological, and biological characteristics related to each patient into a mathematical model could be holistic in terms of probability prediction based on the primary outcome [28, 29].

Differences in median OS depend on the population studied, the stage diagnosed and the treatments received. In our case, the median OS obtained was 934 (95% CI: 694, 1176) days. Based on German data, Hardtstock et al. [30] found that the median OS of NSCLC patients was 351 days. Meanwhile, David et al. [31] found that the median OS for LC patients who had undergone surgery was 9.1 months and 4.2 months for those who had not. Depending on age group stratification; Wu et al. [32] and Torre et al. [33] proved that patients diagnosed over 60 years of age were more likely to be associated with worse survival, which is somewhat contradictory to our results, as the division of age into categories was based on quartile and not risk group stratification.

We should note that not all LC patients can benefit from surgery [34], but the majority of those who do, have undergone radiotherapy, and chemotherapy [35]. Interestingly, however, chemotherapy is not found to be an independent prognostic factor (*p =* 0.8) indicating its little effect on prognosis. For the past 30 years, and based on natural compounds, chemotherapy has been considered as an essential therapy for appropriate LC patients [36], with no proven benefits when is used alone or in patients with fourth stage of the disease, but it may adduce benefits when used in concomitant with radiotherapy, surgery, [37] - and targeted therapy. Performance status (PS), as a subjective composite to evaluate the patient’s wellness, is a key factor reflecting the patient’s ability to carry on normal activities. Several previous studies have reported the role of PS as a prognostic signature impacting the survival rate in different age categories [38–41]. Regarding medical urgency associated with late diagnosis of advanced disease, we found that SVCS, as well as pleurisy syndrome, were all associated with poor survival in patients at the different stage categories of the disease. In a retrospective study conducted by Fahem et al, [42] they concluded that SVCS was a predictive factor for mortality in broncho-pulmonary cancer in addition to pleurisy syndrome. Furthermore, pleurisy syndrome had also an impact on survival when it was associated or developed as a sign of non-response to treatment or simply progression.

Even though literature recognizes the importance of the histological type signature in terms of disease prognostication and impacting survival, [43, 44] we did not find any convergence to with the literature when differentiating the disease categories by dividing into epidermoid cancinoma, neuroendocrine carcinoma, adenocarcinoma, adenosquamous carcinoma, and small cell carcinoma, and taking adenocarcinoma as the reference. Based on the IASLC paper, which indicates among all the histological subtypes of LC, adenocarcinoma remains the more favorable prognostic predictor than the other subtypes [45]. Furthermore, several studies have found, based on different types of analyses, depending on the objective element of the study, that histology type is an independent prognostic predictor and have therefore been integrated to construct the nomogram [35, 46].

We decided to exclude both clinical M category, and comorbidity variables from subsequent MVCA because they would have a bad impact on the total assigned model by being biased, even if they were significant in the results and had been declared independent prognostic factors.

We did not add stage at diagnosis into the Cox analysis for the straightforward reason that stage is mirrored by the combination of T, N, and M categories, and when it is included in the analysis, it results in a substantial bias in the model without any relevance due to information redundancy.

To the best of our knowledge, this is the first nomogram for predicting survival for patients diagnosed with LC based on a North African cohort and long-term follow-up, reflecting the characteristics of the African population in terms of disease response and survival.

However, the creation of clinical prediction models is more significant for enhancing patient prognosis when compared to the analysis of independent risk factors. More importantly, all of the indicators used in this study can be acquired and determined clinically. As a result, the model has improved prediction capabilities and increased dependability, making it a useful tool for clinical decision-making, risk assessment, and patient consultation. This scoring system should make it easier for doctors to deal with these problems. Additionally, this tool might offer data for patient categorization in clinical research design, thereby improving comparability between study arms. Compared to the TNM staging system and certain previous prognostic models, we believe the developed nomogram provides more accurate results.

We should note that this study contains certain limitations. First, this tool needs to be externally validated by an African cohort to make sure the prognostic factors are the same across the continent. Second, due to lack of access to emerging technologies, some molecular aberrations such as EGFR mutation, ALK-EML4 fusion, PDL-1, ROS1, mTOR, are not included in the study as they are not routinely requested until the end of thelast year (2021). Third, our model is still limited by the nature of retrospective data and inability to extract convenient parameters such as vascular invasion, perineural invasion, and lymphatic permeation. Fourth, the patient’s medical records do not contain information on systemic treatments, including type of surgery and radiation dose. To enhance this model, extra work should be done on prospective data gathering, patient follow-up, expanding the recruitment area, and inclusion of additional variables.

## Conclusion

In conclusion, we built a clinical prediction model to determine each LC patient’s unique prognosis. With this tool, clinicians can more precisely predict individual patient survival rates, and treatments strategy. We seek to further develop personalized treatment by conducting quantitative analysis of prognostic-related parameters.

## Data Availability

The datasets used and/or analysed during the current study are available from the corresponding author on reasonable request.

## Abbreviations

OS: Overall Survival
LC: Lung Cancer
NSCLC: Non-Small Cell Lung Cancer
SCLC: Small Cell Lung Cancer
UVCA: Univariate Cox Analysis
MVCA: Multivariate Cox Analysis
SVCS: Superior Vena Cava Syndrome
AUC: Area Under Curve
ROC: Receiver Operating Characteristic
